# Medical Reminiscences of New York

**Published:** 1860-04

**Authors:** D. C. O’Keefe

**Affiliations:** Atlanta, Georgia


### ARTICLE III.

*Medical Reminiscences of New York.* By D. C. O'KEEFE, M. D., Atlanta, Georgia.

### NEW YORK HOSPITAL—SERVICE OF DR. BULKLEY.

*Case* 1.—Ovarian Dropsy in a young woman 30 years old—has been in the Hospital some time, but has had no urgent symptoms, requiring interference, until recently. It is admitted on all hands, that neither diuretics nor any of the

2

usual hydropic remedies, do any good in this species of dropsy. The fluid is contained in a sack or sacks—unilocular or multilocular; this, I think, belongs to the former. There is a sign, it is said, which distinguishes this from other varieties of abdominal dropsy, viz: in this there is dullness over epigastric region; in others, clearness. The sign will not be *always* verified, but is in this instance as compared to a case in another ward, where the effusion is dependent on cirrhosis of the liver. Dyspnœa suddenly set in a few days ago, and in his (Dr. B.'s) absence, his colleague, Dr. Smith, drew off the water, and used Iodine injections afterwards to obliterate the sack, or, rather, to break up its habit of secretion, for it cannot be expected that such a large sack can be obliterated. We defer tapping, in these cases, until obliged by urgent symptoms, to resort to it for their relief. It is sometimes followed by unpleasant consequences, such as peritonial inflammation, and a secretion of pus in the sack instead of serum—in which latter case, irritative fever will be set up, and the patient will sink under it.

He has not been able to see the philosophy of injections in these cases—cannot speak from personal experience, however, on the subject. His colleague, Dr. Smith, who has more experience on this point, thinks well of the operation. The injections are to be repeated after next tapping, if not successful in arresting the secretion in the present instance, which they evidently have not been, for the effusion is accumulating again very fast.

*Case* 2.—Catalepsy in man aged 45 years. Was picked up in the street, and, therefore, his history is unknown.

His symptoms are mixed—is conscious at times, and furious at others—the limbs will remain in any position they are placed in.

*Case* 3.—Epilepsy in man 30 years old—history unknown. Is now in a comatose condition—has not been lucid in three days since his admission—has had several epileptic convulsions. Injections of assafetida are good, and have been used in this case.

*Case* 4.—Peritonitis in a man 40 years old. You recollect his condition one week ago—abdomen hard, painful and lympanitic. Was leeched freely, and put on the opium treatment as recommended by Prof. Clark of this city. He has taken one grain every hour for several days, and this is only a moderate quantity under such circumstances—much more has been given. Prof. Clark is indifferent whether mercury or any local applications be used; opium is the essential element in the treatment. You perceive how this man's symptoms have passed off—he is now convalescent.

*Case* 5.—Acute malarial cacheria. Here is a case of a boy from New Orleans, aged 20 years, who has had intermittent fever, and who is now having abscesses in various parts of the body. There is no local organic disease, but a general poisoning of the system from the malarial influence. Some times this influence will manifest itself in organic derangement—for example, in another ward, there is a case of enlargement of the liver and spleen, and another case of chronic diarrhœa from the same influence. Abscesses occur as they do in other cases of blood poisoning—*e. g.* pyœmia and scrofula. The treatment for them all is a nourishing diet, pure air, quinine, iron, &c., &c.* The case of enlargement of the liver and spleen, from the same cause, in another ward, is very much benefitted by the treatment indicated; the enlargement of the abdomen has diminished three inches, on which was painted the Tr. Iodine. The chronic diarrhœa is but slowly improving, in which opium is used conjointly with the above treatment.

CASE 6.—Hemiplegia in a man 40 years old. This man has had eight apopletic seizures, in two only of which has he lost his consciousness; he has now lost the use of one side, as you perceive, and articulates but imperfectly—is conscious and in his reason. I suspect that syphilis has something to do in the production of this man's trouble.—Dr. Todd says that hysterical hemiplegia can be distinguished

*This boy looks exactly like the cases one sees in South-Western Georgia, known as tallow-faced, dirt-eating subjects.

from hemiplegia dependent on organic cerebral disease by the manner in which the affected leg is moved. In the former, the limb is dragged along the floor in a straight line; in the latter, the limb describes an arc of a circle in each step—it is a circumflex movement. This case, and that of a woman up-stairs, disprove the correctness of this test—the circumflex movement is absent in both, and they are evidently dependent on organic cerebral affection—the woman has facial paralysis which is certain evidence of cerebral lesion.

CASE 7.—Phthisis in a boy 20 years old. This boy has tuberculous cavities in his lungs. One of these tuberculous abscesses has opened into the pleural cavity, giving rise to pleuritic effusion, and the admission of air into the pleura—he has therefore hydro-pneumo-thorax, and his dyspnœa, which is distressing, is caused in this way.

CASE 8.—Billious Remittent Fever in a man 23 years of age, from Aspinwall. Says he was in *perfect* health until noon yesterday, when he was taken sick. This fact of dating invasion to a particular hour, is characteristic of periodic fever; a man having typhoid fever can hardly tell you when he first began to feel unwell. His indisposition came on him imperceptibly—not so with periodic fever—its access is well marked and decided—a sudden change from health to severe sickness.

*Remarks by the writer.*—In this case we recognised the features of an old acquaintance, being the first *well-marked* case of billious remittent fever we had seen in many years. When a medical student in Augusta, Georgia, in 1847–'48, we saw much of this type of fever in the practice of our preceptors (Profs. H. F. and R. Campbell), and at that time the whole subject of malarial fevers was so thoroughly discussed by the able Professor of Practice in the Medical College of Georgia, (Dr. L. D. Ford) that its graduates went forth with their minds full of the subject. The graduates of that period will recollect how large a proportion of the course on practice was devoted to the consideration of malarial fever and its intimate connection with spinal irritation, showing (and justly too) the importance attached to the subject. In 1849, we commenced practice in Green county, 100 miles above Augusta, where the typhoid type of fever prevailed, and here in a practice of eight years, we do not recollect to have seen a single well defined case of the remittent fever, of which for two years we had seen and heard so much in Augusta; and this fact was so striking that we often felt the time and attention bestowed upon the subject while a student could have been more profitably employed. From the attention recently devoted to typhoid fever by the Augusta physicians, especially by our friends, the Profs. Campbell, we presume the same substitution of typhoid for malarial fevers has occurred there that has marked other places. It has been a frequent remark of some practitioners, and especially some of the older members of the profession, that "typhoid" was but a new name for the old bilious fever, and their treatment accorded with their views, but the results of such treatment were so unfavorable that public opinion required its abandonment.

But to return to remittent fever. We have often wondered how remittent and typhoid fevers could be confounded by any one who had seen both diseases, and this case but confirmed our surprise. The very appearance of the two diseases are sufficient to show the difference. Here are two cases lying side by side, in the same ward. One was seized suddenly, at a particular hour of the day, who was previously in the enjoyment of perfect health; he may or not have been taken with a chill, but a fever of a decided character soon succeeds; the skin is flushed and burning hot; pulse full, strong and frequent; violent headache, and pains all over the body, particularly in the back; has nausea and vomiting of bilious matter; his tongue becomes covered with a thick whitish fur, and cannot get a moment's rest.—He has a paroxysm of decidedly sthenic fever that is almost never seen in typhoid fever. In the course of several hours these symptoms moderate; a sweat breaks out, and the patient feels better.

Now take the other case. He can scarcely tell you when his sickness commenced; generally he has been ailing for several days—sometimes a week or two; he gradually lost his appetite, and felt indisposed to perform mental or bodily effort; his fever is moderate, 80 to 110; heat of surface but slightly increased—sometimes not at all; color as often pale as red; has no severe pain anywhere—generally has slight headache; no general suffering and restlessness as in the other case; nausea and vomiting not marked symptoms; sometimes sleeps a good deal—at others, is wakeful, and cannot sleep without opiates; no *decided* exacerbations or remissions, or free sweats, as in the other case. In a word, the diagnosis, to our mind, is easily made out.

CASE 9.—Acute Rheumatism in a man 24 years old. This man has taken three ounces of sal rochelle every 24 hours, for eight days, (this being the usual treatment here, viz: the rochelle salt or some other alkali, in large quantity, until the urine ceases to give an acid reaction) which has not changed the acidity of his urine. It has been discontinued for two reasons: first, that it was doing him no good, and second, because there is some reason to suspect that it is a case of gonorrhœal rheumatism, and therefore put him on the iodide of potassium, on which he is improving.

PROF. ALONZO CLARK'S CLINIQUE.

CASE 10.—Goitre of about one year's standing, in a girl 12 years of age—enlargement of thyroid body. Surgeons some time ago took this disease out of the hands of the physician, and thought they were going to accomplish great things by its removal. They attempted removal, but found so many blood vessels in the way, that at the present day, no one resorts to the operation. So they have given it back to the physicians again, and we cannot do much for it. For our encouragement, however, he was happy to be able to say, that he had seen two cases cured recently. The treatment was commenced early in the history of the enlargement, and consisted in moderate leeching, tincture of iodine locally applied, and he did not recollect whether or not the iodide of potassium was used internally. The leeching was moderate, but long-continued—one or two applied once or twice a week. The application of leeches to this part of the body of a female is a serious matter—the marks of the leech-bites will remain during the patient's life, and therefore the consequences of the remedy should be explained before-hand, in order to avoid blame.

CASE 11.—Chorea in a girl nine years old. When this disease occurs in boys, he is apt to suspect the pre-existence of pericarditis; it very often follows that disease in boys—indeed it is the most frequent cause of it. In girls such relation does not exist between the two diseases. When he sees this disease in a boy, therefore, he suspects two things: first, that he has had pericarditis to which this is a sequel; and second, that the pericarditis itself was the sequel of rheumatism. When a child has rheumatism for four or five days, cardiac trouble is *very apt* to be developed; it may exist two or three days without such development. The seat of chorea is probably in the spinal cord, at least all the knowledge we have on the subject, points to that part as the seat; it is not a fatal disease, and hence the difficulty of ascertaining its morbid anatomy. The vast majority of the cases get well, and when it proves fatal, want of sleep and the violent and continued involuntary muscular efforts are the cause of it. He has seen two such cases.

If your case sleeps, it will not die; but if they cannot sleep, it will wear them out. Treatment very simple. Laxatives to commence with, then iron, and he prefers the old carbonate in ten grain doses three times a day; good air and nutritious diet. With the exceptions above mentioned, he had never seen a case that did not get well on this treatment in four to six weeks.

*Case* 12.—Congenital paralysis of arms in a child eighteen months old. He did not know what it was owing to—whether injury to nerves from malposition of arms in delivery, or defective development of the nerves. You perceive muscular paralysis allows dislocation at the shoulder-joint, forward, backward, and downward, but not upward, on account of bony obstacle. There is danger in such a case as this, of the muscles losing their muscularity—that is, of a change from muscular fibre into imperfect adipose matter—something like what happens to the cadaver in certain kinds of soil, where it is converted into a kind of soapy substance, and preserved from decomposition for a long time. Treatment.—Nothing promises so much as electricity or galvanism; friction with hand may afford electricity enough to do some good, but the daily shocks from the battery will do better. Strychnia also is valuable. These two remedies will preserve the muscularity of the muscles, even if they do not affect their innervation.

*Case* 13.—Paralysis agitans, in a man 49 years old. This disease belongs to old age, and this man is too young to have it from that cause. You perceive he talks well—there is no impediment in his speech—this shows you it is not cerebral in its origin—when of such origin it is almost always attended with imperfect speech. It is a wavy motion of the muscular fibres, which do not act in harmony. Sometimes such a case as this may simulate chorea, but they are easily distinguished—the wild, irregular jerking and tossing of the limbs of chorea do not exist here.

This man has lived temperately and regularly—has not been exposed to lead poison, and he (Dr. C.) did not know how to account for his paralysis agitans. It is no use to reason out a cause—had as well say, at once, we don't know.—He could appreciate what he discovered with his senses, but in medical matters, the less we reason, in his opinion, the more we know—confine ourselves to facts as much as possible. Treatment.—Oxide of zinc, nux vomica and iron.

DR. CONANT'S CLINIQUE AT NEW YORK MEDICAL COLLEGE.

*Case* 14.—Pelvic Abscess in woman 30 years old. Ovarian inflammation is more frequent in left than right ovary—

three out of four is said to be the relative proportion, and there is an anatomical reason for it. The venous blood from left ovary is returned to the heart by the emulgent veins, but from the right the ovarian veins open directly into the vena cava ascendeus, and hence, congestions and inflammations are much less apt to occur in right than left ovary. In these cases the cellular tissue of the pelvic cavity becomes infiltrated, and a hardened mass is the result, which you can feel through the vagina as well as the abdominal walls. In this case, the abscess opened into the vagina, and discharged for months, and confined the patient to her bed; but now the discharge has ceased, and the opening in the vagina has healed up, and yet she suffers from uterine symptoms. There is still induration and enlargement of the pelvic tissues, and the uterus will now be cupped, with uterine cupping apparatus, with the view of relieving the congestion.

*Case* 15.—Fibrous tumour in anterior wall of uterus—woman about 35 years old. Presented several specimens of dried uteri with fibrous tumours—are extremely common in negroes, especially mulattoes; you hardly ever find a mulatto who has a sound uterus—all these specimens (five or six in number) were obtained at "The Colored Home."—They may be sub-mucous or sub-peritomal—when the former they give much trouble; sometimes cause profuse hemorrhage at menstrual periods, and most always cause abortion.

When sub-peritoneal, *i. e.*, outside the muscular coat, the uterus may enlarge and give birth to a fœtus at full term.—They may be felt sometimes through vagina, and sometimes through abdominal walls, but best from both places. He had succeeded sometimes, very satisfactorily, in reducing their size, and removing them even, by the internal use of the iodide of potassium, and the ointment of the same, applied locally, to the tumor over abdomen or through vagina, or both, according as it may be convenient. Had a case under treatment now, that has been reduced in this way, from a large size down to the size of a filbert. The magneto-galvanic current will sometimes promote absorption, which will now be used in this case, applying one pole to os uteri, and the other to tumor over abdomen. (The apparatus for applying the current to the os uteri, was a piece of common wire, having a loop at each extremity, and its body enveloped in a gum elastic bougie to prevent loss of current; one of the loops was large, and slipped over the handle of the pole; the other was small, to apply to the os uteri.) The current should be applied for five minutes at first, and increased gradually, until half an hour is reached.

CASE 16.—Retroflection in a young woman 21 years old. This young woman has been married a few months, and her ill-health dates with her marriage—sexual intercourse has always been painful to her. Her uterus is now reduplicated backwards. Restore the uterus to its proper position with the uterine sound, and then if it shows any tendency to return, use his (Dr. Conant's) intra-uterine pessary to maintain it in situ. The sound should be used with care—had seen pelvic inflammation, metritis, &c., follow its use—still, if used with care, no trouble will be apt to follow. In this case, the replacement of the uterus (the fundus of which can be distinctly felt in the vesico-vaginal cul-de-sac) by Professor Peaslee, several weeks ago, was followed by a painful condition of the pelvic viscera, so much so that the patient had to keep her bed for a week.

The fundus has fallen back again, but in consequence of a dysentery under which she is laboring at present, we will not attempt to restore it. The sound should not be used when there exists any pelvic irritation. His intra-uterine pessary is made of galvanized rubber, and answers better for flexions of the uterus than any instrument he had ever used—every one, however, thinks his own instrument the best—had used it now in ten or eleven cases, and had never known any inconvenience follow its use—had allowod it to remain in one case two months without the patient suffering any inconvenience from it. Indeed she could not tell she was wearing it, except when fatigued; she could then feel its very shape, as she alleged. It is very durable and does not soil.

CASE 17.—Dysmenorrhœa in a woman 35 years old. This woman was here last week, and you may recollect her history. Has been married nine or ten years without children—is a stout, hearty Irish woman, and has a healthy husband—has suffered very much at monthly periods—her os tincæ is small and circular like a virgin's—could not introduce the point of Sympson's uterine sound into it. On a former occasion, he had scarified the os slightly, and she reports that she has not suffered near so much during the menstrual period which she has passed since that operation. We will now introduce a small sound—there you see it in the uterus—will now introduce a larger one, and then use this dilator to dilate the cervix, (which was done to a considerable extent, the patient making a due amount of fuss in the meantime.) The menstrual discharge escaped through this narrow passage with great difficulty—the uterus contracts and tries to expel it, and this gives rise to painful menstruation. Neither could spermatozoa find their way into the uterus through such a cervix as this, which, in all probability, may account for her sterilility.

DR. MARCOE'S CLINIQUE.

CASE 18.—Alopecia in yonng man—is completely bald—cannot promise much in this case—is almost incurable—may use remedies that are known to promote the growth of the hair, of which this is his favorite:

℞.—Tr. Canthar.

Tr. Capsici.

Aquæ Cinam., *aa* ℥i.

Spts. Lavend. Co., ʒij.

Of this mixture use one part to two of cologne-water rubbed on with a brush.

CASE 19.—Eczema Impetigenodes of scalp in a little girl—a combination of a vesicular and pustular disease. The scab of eczema is thin and whitish; that of impetigo thick and greenish. *Treatment*—Poultice for a few days to remove the scabs, and then Goulard's Cerate.

CASE 20.—Internal bleeding hemorrhoids in man 40 years old. Straining as if at stool, forced the tumors, three in number, out so that they could be seen. They are deprived of epithelium, and hence the hemorrhage. *Treatment*—Cauterize each tumor separately, with the pure nitric acid, and wash the parts afterwards. Has found this course of treatment almost a certain cure. If the tumors are external, they may be snipped off and then cauterized as above.

CASE 21.—Simple Conjunctivitis. Don't use astringents in such a case as this at first, they will make it worse ; rather use warm water bathing, and ointment to prevent lids from sticking, and if the case is not well in three or four days, then apply astringents.

PROF. ALONZO CLARK'S CLINIQUE.

CASE 22.—Here is a specimen of diabetic urine, which, on standing, shows the presence of a crust on its surface, which is called the yeast plant or torula, an extractive substance of a vegetable character, which, through some fault in the digestive process, is not assimilated, but finds its way out of the body through the kidneys. This is different from that other vegetable substance—the penicilium glaucum—to which he has called our attention, and its presence in the urine indicates a different state of things. The difference between them is indicated by their microscopic characters which are distinct and well marked. This indicates the presence of sugar in the urine, and is a more reliable test than any other that I know of; Trommer's test for sacharine urine is not so reliable as this : has been applied to this specimen, but does not give satisfactory results. *Treatment*—Anecdote—An old doctor in the city used to ask him, every time they met, with uplifted hands, "in the name of God, doctor, do you know what will cure diabetes?" He (Dr. C.) was disposed now to ask the same question—thought a few years ago, he had *cured* two cases, and so reported to the New York Medical and Surgical Society ; but he was sorry to add, that this urine which he had here, was from one of them. The other case might be considered as having been cured; he died of pneumonia six years after cure, and was free from the disease during that time, so far as he knew.—The case from which this specimen was obtained is a physician, and he considered himself well for six or eight months—this is what he had supposed was the second case, but it is not yet cured—at the end of the time stated, the disease returned. The treatment of these two cases consisted of carb. soda, ten to twenty grains, five or six times a day; diet, meat and crackers; no bread or fermented food of any kind; no potatoes—succulent vegetables, such as onions, celery, &c., were used; also exercise, and counter-irritation to back of neck. Under this treatment this physician got well, and remained so eight months; and the disease is kept in check now under such management. By it the quantity of urine was diminished from two or three gallons a day to two quarts, and its sp. gr. from 1060 to 32. He gained flesh also: he still has confidence in the treatment, and thinks he will get well. He knows of some eight cases controlled in this way, by the course indicated, but none cured.

CASE 23.—Abdominal tumor in woman 30 years old. Her complexion is florid, and she is not much debilitated; was delivered of a full grown, healthy child, fourteen months ago; was larger than usual during her pregnancy, and thinks the tumor commenced during that time; the enlargement did not entirely disappear after delivery. Now, what is the seat and character of this tumor? And first, as to its seat: You perceive percussion is dull over it; it gives no more resonance than the thigh does; if it were ascites and the collection no greater than it is here, the intestines would not all be crowded back by the fluid; some would be in contact with the abdominal wall, and we would get intestinal resonance. We will therefore exclude ascites. Is it in the cavity of the uterus? No; because this tumor is partly fluid and partly solid; part of it is a hard, solid substance, probably fibrous, and part (the larger) is fluid, as shown by distinct fluctuation. You do not have tumors of this kind in the uterus; you may have collections of fluid in the cavity of that organ, but its walls are so thick you will not get fluctuation. Here fluctuation is manifest, and therefore the uterus is not its seat. We have mixed tumors of this character in the ovary, and this, he thinks, is clearly an ovarian tumor. The ovarian cyst rises up in front of the intestines and pushes them back, and therefore you cannot get intestinal resonance. Having made out the diagnosis, you will want to know something about the treatment, and this may be told in few words, viz: "hands off." A suspensory bandage will afford some comfort; don't tap until it is rendered necessary by dyspnœa; then, and not before, is it justifiable. Attempts at radical cure, such as injections, &c., have been made, but have not given satisfactory results. Nature sometimes effects a cure by the tumor opening externally, and discharging its contents, then pus is secreted by the lining membrane, and discharged externally, and this process goes on, the cavity contracting until it is finally obliterated. Dr. Kimball, of Lowell, has imitated nature in this plan by producing an artificial opening into the sac, by means of escharotics; but his patient sank under the suppurative drain which followed. The removal of these tumors has been proposed and accomplished, but not with encouraging results. Of the *selected* cases operated on, half died; this is too serious a responsibility to be assumed. Dr. Kimball has operated fifteen or sixteen times with about such results as I have stated. He has abandoned the operation now, and will not perform it if he can avoid it. Prof. Peaslee of this city, has removed both ovaries, and Dr. Kimball operated on one case twice, and the woman got well. Dr. Atlee, of Philadelphia, has performed the operation several times, and is a strong advocate for it. They had better be left alone, in my opinion.

Case 24.—A child two years old, is partially idiotic, following a three days' attack of convulsions, which occurred eight months ago, at which time it lay in bed for a month in a state of unconsciousness; it could not see until recently.

He thinks this was a case of acute hydrocephalus, from which the child has recovered, (a very rare occurrence, one in eight or nine,) and a consequence of which is the idiotcy. He thinks it a promising case: that in the course of half a year, or a year, the child can talk and walk, and use its other faculties. He would not do much for it: would give it iod. potass. as an alterative and diuretic, on alternate weeks: by intermitting in this way, its use and good effects can be kept up for a long time.

CASE 25.—A girl aged 16, has anemia and cardiac affection with bruit de diable (free translation, Devil of a sound), has also icthyosis, a rare form of cutaneous disease which differs from psoriasis, in the former having the skin thickened, the latter not. *Treatment*—Iron, regimenial management, good diet, &c.

CASE 26.—A child 6 or 8 years old, is very much emaciated—has marasmus or phthisis, which terms literally, mean a wasting away. Marasmus, however, we use to designate the emaciation attendant on a diseased condition of the mesenteric glands, which prevents the entrance into the system of the oily portions of the food. Phthisis means the emaciation attendant on the deposit of tubercles in the lungs; and tubercular or chronic peritonitis is the name given to it when the tubercles are deposited on the peritoneum. In the case before us the emaciation is due to tubercular peritonitis. His abdomen is *prominently* enlarged—has *permanent* tympanitis, and this is a diagnostic mark of the disease. This trouble has followed measles.

CASE 27.—Two cases of carcinoma of the stomach; one in an Irishman 50 years old, the other a German 35 years old. The latter is a potter, works in earthen ware, some of which is lined inside with lead. This you will do well to bear in mind, that the glazed earthen ware has a lining of lead upon it which may give rise to poisoning if such ware be used in cooking, and especially if an acid come in contact with the leaded surface. This kind of ware, on account of its cheapness, was used in cooking at the West, a few years ago, and caused extensive lead-poisoning. But in the cases before us, the symptoms are emaciation, vomiting daily, in one, occasionally in the other; pain, and a sense of swelling in the epigastric region—the latter, probably, caused by gaseous distention. There is a well-defined tumor in both, larger in the old man. Now, to what is this condition of things owing? You may have their explanations of it, viz: Carcinoma, fibrous degeneration and ulcer. He thinks all the symptoms point, with tolerable accuracy, to the first. Well now, we have made out the diagnosis, and *cui bono;* one advantage you have gained, is, that you know what you have got to treat, and you will not be trying to accomplish what you cannot. These cases are considered some of the *opprobria medicorum*, but, as in many other conditions, we can prolong life and mitigate suffering. Prussic acid, one or one and a half drops, three times a day, will sometimes relieve the vomiting—give them the oily food, cream, butter, cod-liver oil, &c.; iron and opium, if much pain exist. He uses the camphorated oil over the stomach as a counter-irritant—they say they feel better under its use.

